# A novel technique for the single-port laparoscopic percutaneous extraperitoneal closure (SLPEC) of paediatric isolated giant inguinal hernias using double-modified hernia needles

**DOI:** 10.1038/s41598-024-60476-x

**Published:** 2024-07-04

**Authors:** Long-Yao Xu, Xu Cui, Wen-Hua Huang, Liu Chen, Chao-Ming Zhou

**Affiliations:** Department of Urology, Fujian Children’s Hospital (Fujian Branch of Shanghai Children’s Medical Center), College of Clinical Medicine for Obstetrics and Gynecology and Pediatrics, Fujian Medical University, No. 966 Hengyu Road, Fuzhou, 350000 China

**Keywords:** Giant inguinal hernia in children, Single-port, Double-modified hernia needles, Laparoscopic high ligation of the hernia sac, Hydrodissection, Diseases, Gastroenterology, Health care, Medical research, Signs and symptoms, Urology

## Abstract

The objective was to explore the efficacy of single-port laparoscopic percutaneous extraperitoneal closure using double-modified hernia needles with hydrodissection (SLPEC group) and two-port laparoscopic percutaneous extraperitoneal closure (TLPEC group) for the treatment of giant indirect inguinal hernias in children. We performed a retrospective review of all children with giant indirect inguinal hernias (inner ring orifice diameter ≥ 1.5 cm) who underwent laparoscopic high ligation of the hernia sac at FuJian Children’s Hospital from January 2019 to December 2021. We collected data from the medical records of all the children and analysed their clinical characteristics and operation-related and follow-up information. Overall, this study included a cohort of 219 patients with isolated giant inguinal hernias who had complete clinical data and who had undergone laparoscopic high ligation of the hernia sac at our centre. All procedures were successfully performed for the 106 patients who underwent SLPEC and for the 113 patients who underwent TLPEC at our centre. There were no statistically significant differences in patient age, sex, body weight, follow-up time or the side of inguinal hernia between the SLPEC group and the TLPEC group (P = 0.123, 0.613, 0.121, 0.076 and 0.081, respectively). However, there were significant differences in the bleeding volume, visual analogue scale (VAS) score, and postoperative activity time between the two groups (P ≤ 0.001). The operation times in the TLPEC group were significantly longer than those in the SLPEC group (P = 0.048), but there were no significant differences in hospital length of stay or hospitalization costs between the two groups (P = 0.244 and 0.073, respectively). Incision scars were found in 2 patients in the SLPEC group and 9 patients in the TLPEC group, and there was a significant difference between the two groups (P = 0.04). However, the incidence of ipsilateral hernia recurrence, surgical site infection, suture-knot reactions and chronic inguinodynia did not significantly differ between the two groups (P = 0.332, 0.301, 0.332 and 0.599, respectively). Postoperative hydrocele occurred in only 1 male child in the SLPEC group and in no male children in the TLPEC group, and there was no difference between the two groups (P = 0.310). In this study, there were no cases of testicular atrophy or iatrogenic ascent of the testis. Compared with the TLPEC group, the SLPEC group had the advantages of a concealed incision, light scarring, minimal invasiveness, a reduced operation time, minimal bleeding, mild pain and rapid recovery. In conclusion, SLPEC using double-modified hernia needles with hydrodissection and high ligation of the hernia sac is a safe, effective and minimally invasive surgery. The cosmetic results are impressive, and the follow-up results are promising.

## Introduction

Although the incidence of giant inguinal hernia is not high, the diameter of the hernia ring, repeated friction, or incarceration of the inner ring can easily cause oedema and the formation of many folds in the hernia sac, which greatly increases surgical difficulty and the risk of recurrence^1^. Traditional high ligation of the hernia sac can effectively improve the clinical symptoms of children, but it is not conducive to improving the prognosis of children with more severe body damage, more bleeding or a slow postoperative recovery^2^. High ligation of the hernia sac using an epidural needle with the assistance of single-port laparoscopy has been widely and successfully applied in various countries^3^. Wang et al.^4^ conducted a retrospective analysis of 1142 patients with paediatric inguinal hernia from our hospital and found that laparoscopic percutaneous extraperitoneal closure of the internal ring using an epidural needle is a safe and feasible method for the treatment of inguinal hernias in children. This method has the advantages of less trauma, no scarring and a good cosmetic effect. With the widespread use of hernia needles in the clinic, high ligation of the hernia sac under laparoscopic guidance without injury caused by hernia needles has been gradually applied in the clinical treatment of indirect inguinal hernia in children. This operation has the advantages of simplicity, a small incision, less intraoperative blood loss, and rapid postoperative recovery^5^. Separation of the peritoneum by water injection is simple, safe and effective^6^.

However, due to the small sample size and short follow-up time in previous studies, no systematic studies on the treatment effect of high ligation of the hernia sac and the associated rate of recurrence of giant inguinal hernias have been conducted. Therefore, 219 children with giant inguinal hernias admitted to our hospital between January 2019 and December 2021 were retrospectively analysed in this study. Single-port laparoscopic percutaneous extraperitoneal closure (SLPEC) for high ligation of the hernia sac using double-modified hernia needles combined with the hydrodissection technique and two-port laparoscopic percutaneous extraperitoneal closure (TLPEC) for high ligation of the hernia sac were the procedures employed. The objective of our study was to evaluate the efficacy of SLPEC combined with water injection for high ligation of the extraperitoneal hernia sac in the treatment of giant inguinal hernias in children.

## Results

### Patient characteristics

Twenty patients without complete clinical data and 10 patients with incomplete follow-up data were excluded. This left a cohort of 219 isolated giant inguinal hernia patients with complete clinical data who underwent laparoscopic high ligation of the hernia sac at our centre. All procedures were successfully performed for 106 patients who underwent SLPEC and for 113 patients who underwent TLPEC at our centre.

There were no statistically significant differences in patient age, sex, body weight, history of acute incarceration, follow-up time or side of the inguinal hernia between the SLPEC group and the TLPEC group (P = 0.123, 0.613, 0.121, 0.115, 0.076 and 0.081, respectively). Patient characteristics are described in Table 1.

**Table 1:** Clinical characteristics of the SLPEC and TLPEC groups before operation.

Characteristics	SLPEC	TLPEC	t (χ2) value	P value
Age (years) (Mean ± SD, range)	3.58 ± 2.65, (0.66–11.00)	3.04 ± 2.65, (0.12–12.00)	T = 1.549	0.123
Gender, n (%)			χ^2^ = 0.255	0.613
Boys	81(76.4%)	83(73.5%)		
Girls	25(23.6%)	30(26.5%)		
Side, n (%)			χ^2^ = 5.035	0.081
Right	67(63.20%)	57(50.4%)		
Left	38(35.8%)	56(49.6%)		
Weight (kg) (Mean ± SD, range)	16.90 ± 7.56, ( 8.00- 35.70)	15.31 ± 7.50, ( 4.00- 39.00)	T = 1.559	0.121
History of acute incarceration, n (%)			χ^2^ = 2.488	0.115
Yes	1(0.9%)	5(4.4%)		
No	106(91.1%)	108(95.6%)		
Follow up time (months) (Mean ± SD, range)	19.81 ± 6.03, (12.00–30.00)	18.40 ± 5.58, (12.00–30.50)	T = 1.786	0.076

Single-port laparoscopic percutaneous extraperitoneal closure for high ligation of the hernia sac using double-modified hernia needles combined with the hydrodissection technique (SLPEC group) and two-port laparoscopic percutaneous extraperitoneal closure for high ligation of the hernia sac(TLPEC group). Categorical variables are represented as number (%) and continuous variables as mean ± standard deviation (range).

The bleeding volume, operation duration, postoperative activity time, VAS score and length of hospitalization of the two groups are shown in Table 2. There were statistically significant differences in the incision length, bleeding volume, operation time, VAS score, and postoperative activity time between the SLPEC group and the TLPEC group, while there were no significant differences in hospital length of stay or hospitalization costs between the two groups.

**Table 2:** The intraoperative and postoperative conditions were compared between the SLPEC and TLPEC groups.

Characteristics	SLPEC	TLPEC	t (χ2) value	P value
Operation time (minutes), Mean ± SD (range)	28.36 ± 10.74, (10–70)	31.35 ± 11.44, (15–95)	T = − 1.989	0.048
amount of bleeding (ml), Mean ± SD (range)	0.95 ± 0.57, (0–2)	1.09 ± 0.45, (0–2)	T = − 2.024	0.044
Postoperative activity time (h) Mean ± SD (range)	3.85 ± 0.58, (3.00–6.00)	4.24 ± 0.53, (3.00–6.50)	T = − 5.095	< 0.001
Time of hospital stay (day)	1.93 ± 0.54, (1.3–5.0)	2.00 ± 0.40, (1.3–4.0)	T = − 1.168	0.244
hospitalization costs (yuan), Mean ± SD (range)	8816.18 ± 427.67, (8003.05–9786.39)	8923.70 ± 410.65, (7803.86–9611.66)	T = − 1.800	0.073
VAS score, Mean ± SD (range)	0.59 ± 0.14, (0–4)	1.00 ± 0.94, (0–4)	T = − 3.499	0.001
Incision length (cm)	0.48 ± 0.04, (0.38–0.55)	0.69 ± 0.05, (0.55–0.80)	T = − 35.695	0.001

Single-port laparoscopic percutaneous extraperitoneal closure for high ligation of the hernia sac using double-modified hernia needles combined with the hydrodissection technique (SLPEC group) and two-port laparoscopic percutaneous extraperitoneal closure for high ligation of the hernia sac(TLPEC group). Categorical variables are represented as number (%) and continuous variables as mean ± standard deviation (range).

**Figure 1:**
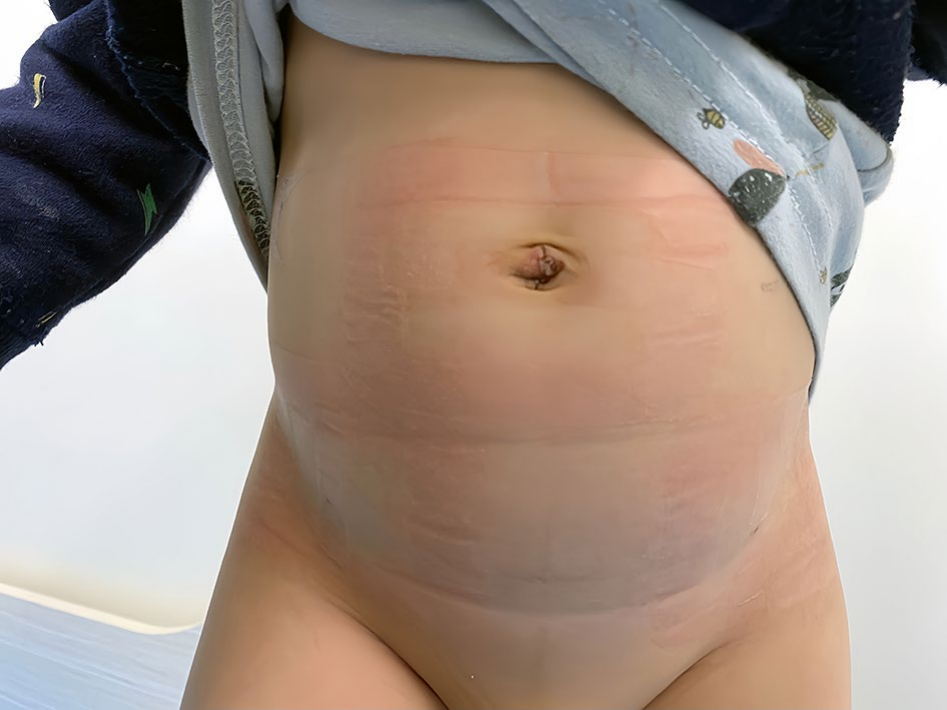
The incision appearance of laparoscopic inguinal hernia repair after 1 month.

The incidence rates of hernia recurrence and postoperative complications in the SLPEC group and TLPEC group are shown in Table 3. In this study, most of the SLPEC group had no scars after 1 month (Fig. 1). Incision scars were found in 2 patients in the SLPEC group and 9 patients in the TLPEC group, and there was a significant difference between the two groups (P = 0.04). There was no significant difference in the incidence of ipsilateral hernia recurrence, surgical site infection, suture-knot reactions or chronic inguinodynia between the two groups (P = 0.332, 0.301, 0.332 and 0.599, respectively). Postoperative hydrocele occurred in only 1 male patient in the SLPEC group and in no male patients in the TLPEC group, and there was no difference between the two groups (P = 0.310). In this study, there were no cases of testicular atrophy or iatrogenic ascent of the testis.

**Table 3:** Postoperative complications in the SLPEC and TLPEC groups.

Complications, n (%)	SLPEC	TLPEC	t (χ2) value	P value
Scar, n (%)	2 (1.9%)	9 (8.0%)	χ^2^ = 4.235	0.04
Ipsilateral recurrent hernia (over total number of hernias), n (%)	0 (0%)	1 (0.9%)	χ^2^ = 0.942	0.332
Surgical site infection	1 (0.9%)	0 (0%)	χ^2^ = 1.071	0.301
Hydrocele (male)	1 (1.2% )	0 (0%)	χ^2^ = 1.031	0.310
Iatrogenic ascent of the testis (male)	0	0	–	–
Testicular atrophy (male)	0	0	–	–
suture knot reaction	0 (0%)	1 (0.9%)	χ^2^ = 0.942	0.332
Chronic inguinodynia	1 (0.9%)	2 (1.8%)	χ^2^ = 0.277	0.599

Single-port laparoscopic percutaneous extraperitoneal closure for high ligation of the hernia sac using double-modified hernia needles combined with the hydrodissection technique (SLPEC group) and two-port laparoscopic percutaneous extraperitoneal closure for high ligation of the hernia sac(TLPEC group). Categorical variables are represented as number (%) and continuous variables as mean ± standard deviation (range).

## Discussion

Inguinal hernia is a common disease in children because the peritoneal sheath does not close or atresia occurs during the growth process^7,8^.A large inguinal hernia is defined as an internal annulus greater than 1.5 cm in diameter^9^. Although the incidence of giant inguinal hernias is not high, the diameter of the hernia ring, repeated friction, or incarceration of the inner ring can easily cause oedema and the formation of many folds in the hernia sac, which greatly increases surgical difficulty and the risk of recurrence^1^. To date, the recurrence rate of hernias after simple laparoscopic high ligation of the hernia sac is still between 0.3% and 10.9%^10^, and it is believed that the recurrence factors include a large diameter before and after opening of the inner ring, a lack of accurate high ligation, and older age (the surgical recurrence rate in adolescents aged 14–18 years is greater than that in children)^11^.

Although many efforts, such as cauterization of the sac, including the medial or lateral umbilical ligament, to repair and excise the sac, have been made to reduce the risk of recurrence^12–14^, all techniques have the disadvantages of complicated operations and great trauma. Marcy was the first surgeon to describe transversalis fascia repair of the internal inguinal ring with interrupted catgut sutures; this procedure was performed for the first time in 1871^15^. In this procedure, the internal spermatic fascia (ISF), an evagination of the transversalis fascia, was incised circumferentially at the internal ring for closure with interrupted sutures. Conversely, attention was given to preserving as much of the ISF tissue as possible; in the study conducted by Yokomori, K, the hernia sac was ligated together with the ISF, as a tougher monolayer, effectively narrowing the internal ring by tightening the neck of the "sleeve" to reduce the risk of recurrence^16^. However, traditional open repair of the anterior and posterior walls of the groin results in a large wound surface area, damages the structure of the inguinal canal, and increases the probability of injury to the lower abdominal blood vessels, spermatic cord and vas deferens^17^. With the development of minimally invasive technology, laparoscopic high ligation of the hernia sac has been used in clinical practice. It has been reported that there is a greater recurrence rate after high ligation of the internal ring orifice^18^, and laparoscopic internal ring closure plus an ipsilateral medial umbilical fold flap (MUFF) cover is used for children with large indirect inguinal hernias to reduce the risk of recurrence; however, this approach may increase the risk of injury to subabdominal blood vessels^13^.Nevertheless, laparoscopic closure of the internal annular opening can also achieve good results in infants with oblique inguinal hernias larger than 2 cm. Among the patients with scrotal oedema in this study, no haematoma or hernia recurrence was observed^19^. This surgery is widely used in the clinic because it does not require dissection of the groin and is associated with less surgical trauma^20^. In addition to improvements in suture techniques, further studies on ligation sutures have shown that compared with single-strand absorbable suture ligation, double-strand nonabsorbable suture ligation can greatly reduce the risk of giant inguinal hernia recurrence^9,21^.Therefore, laparoscopic high-ligation hernia sacs generated by double nonabsorbable sutures were used to treat isolated giant inguinal hernias, and our study achieved good results. The main reason is that the second knot strengthens the first knot and reduces tension, thereby preventing the knot from breaking and loosening and greatly reducing the risk of recurrence. At the same time, the ligature knot is located in the episperitoneal space under the abdominal wall muscle layer, rather than the traditional laparoscopic suture, for which the knot is fixed under the skin, and ligation is more reliable. It was reported that the relapse time is usually within 6 months after the original surgery, when the scar has softened; the age at relapse is often during the preschool period, and the probable contributing causes included restless activity and weak inguinal muscles at this age^22^.Hence, all children were followed up for at least 1 year in this study. There was almost no ipsilateral hernia recurrence in the SLPEC group; however, the ipsilateral hernia recurrence rate was 0.9% in the TLPEC group because of the large diameter before and after the opening of the inner ring.

With the rapid development of endoscopic technology, laparoscopic surgery has become widely accepted by paediatric surgeons because it can reduce obvious skin incisions and improve the aesthetic effect of traditional surgery^23^. Single-port laparoscopic high ligation of the hernia sac is the preferred surgical method for the treatment of oblique inguinal hernias in children^24^. In addition, several efforts have been made to reduce the number of trocar and port site scars and to improve cosmesis, and some related research has led to improvements in the use of hernia needles, from traditional ordinary taper needles and epidural needles to the development of two-hooked core needles and to the latest Amity fascia closure to improve hernia needles^4,25,26^. In particular, Huang et al*.*^26^ reported that SPLPEC of inguinal hernias using a “two-hooked” core needle apparatus in children is a feasible and reliable minimally invasive procedure. It has the advantages of a short operating time, low complication rate, low recurrence rate and good cosmetic results. Our study differed in that our double-modified hernia needles were sourced from the aid fascia closure device, which was produced by Xiamen Surgaid Medical Equipment Co., Ltd., of China (Patent No. ZL 2013 20013865.2). In contrast to the two-hooked core needle used in Huang et al.'s^26^ study, the double-modified hernia needle has two grips, which can act as an auxiliary grip. Moreover, we target isolated giant hernias, which often require auxiliary forceps due to the presence of many folds. In our study, double-modified hernia needles were used to treat giant inguinal hernias. One hernia needle was used as an auxiliary forceps to reduce the number of trocars, increase the flexibility of the operation process and prevent damage to the spermatic cord, vas ductus and peritoneum, which would be impossible to achieve with the two-hooked core needle used in Huang et al.'s^26^ study. In addition, our study included a control group of patients who underwent two-port laparoscopy, while Huang et al*.*'s^26^ study did not include a control group. The results of the present study showed that compared with two-port laparoscopy, single-port laparoscopy yielded good results, was completely effective and had aesthetically pleasing results. Similar to the findings of Wang et al.^4^, there were almost no surgical scars in the SLPEC group because the umbilicus was a hidden natural scar, but 8.0% of patients in the TLPEC group had surgical scars. Notably, the difference in the operation time between the SLPEC and TLPEC group (28 and 31 min, respectively) was statistically significant but not was clinially significant. The reason may be that different surgeons have different levels of proficiency. Compared with TLPEC, SLPEC has no significant advantage regarding operation time; however, the he postoperative activity time, VAS score, incision length and amount of bleeding in the SLPEC group were significantly better than those in the TLPEC group.

It has been shown that hernia stratification impacts the management and prognosis of patients^27^. The European Hernia Society published a new classification of inguinal hernia based on inner ring size in 2018^28^ as follows, highlighting the significance of inner ring size: type 0, no hernia detectable; type 1, < 1.5 cm (or one finger breadth); type 2, < 3.0 cm (or two finger breadths); and type 3, > 3.0 cm (more than two finger breadths). This surgical classification method is complex and limited in its clinical application. The following simple classification methods based on the diameter of the inner ring are accepted by most researchers in China^9^: giant type (inner ring orifice diameter ≥ 1.5 cm), ordinary type (inner ring orifice diameter of 0.5–1.5 cm) and hidden type (inner ring orifice diameter < 0.5 cm). In our study, a large inguinal hernia was defined as an internal annulus greater than 1.5 cm in diameter.

Based on our accumulative experience, the advantages and techniques of this operation can be highlighted as follows:

The internal ring suture is placed in an extracorporeal manner, which conforms to the operative technique employed by most surgeons and thus dramatically shortens the learning curve of this procedure.

The tip of the needle is sharp, and the body is round in shape, allowing the needle to easily penetrate tissues with minor abrasions or cuts. Moreover, the clamp can easily grab the suture and act as an auxiliary forceps to flatten the sac. With a giant inguinal hernia sac (> 1.5 cm) containing many folds, it is difficult to ligate the hernia sac completely. Due to peritoneum weakening and close adherence to the spermatic cord and vas deferens, surgery often leads to peritoneum perforation, bleeding, and damage, particularly to the spermatic cord and vas deferens. Therefore, surgeons often need to rely on auxiliary forceps to assist in performing high ligation of the hernia sac^29^. Using another hernia needle, the fold of the inner ring orifice is grasped to pull and flatten the sac to help smooth the passage of the peritoneal fold and complete the ligation of the inner ring orifice through only a needle-sized incision.

One of the main challenges is to achieve complete circumferential closure of the sac at the level of the internal ring without injuring the adjacent vas deferens or spermatic vessels. The previous surgical methods involved separating the spermatic cord and vas deferens from the peritoneum through auxiliary grasping, improved stitching (e.g., jumping-suture technique, internal orifice circumferential suture method) and the operator’s experience in preventing damage to the spermatic cord and vas deferens^25,30,31^. However, the hernia contents stimulate the neck of the hernia sac, which causes peritoneal inflammation, thickening and adhesion of the inner ring opening. Peritoneal separation of the ordinary hernia needle cannot be accurate to the peritoneal level. When it is difficult for the hernia needle to pass through the peritoneum, the repeated use of auxiliary forceps can easily lead to a small peritoneal perforation or tear, and the peritoneum may be weak after ligation; this may lead to the risk of recurrence due to increased abdominal pressure and increased rupture^32^. Schier^31^ employed interrupted stitches and avoided placing the stitches too close to the vas deferens and testicular vessels. A distance that is too great can lead to potential space for recurrence.

Water injection can reduce damage to the spermatic cord and vas deferens. In an effort to separate these structures from the peritoneum before passing a suture around the base of the sack, Muensterer and Georgeson^33^ developed the hydrodissection-LASSO technique, which is performed using a 22-gauge needle. However, peritoneum separation and hernia sac ligation cannot be accomplished simultaneously in this way. Although Kang and Liu^32^ developed his own water separation needle to achieve certain effects, the needle could still not meet the ligation range due to limitations of the needle length and angle. The application of the water separation process does not require much water, is easy to follow, and does not increase the rate of postoperative recurrence^34^. In our study, by using a needle inserted percutaneously over the internal inguinal ring, saline was injected into the subperitoneal plane circumferentially, the peritoneum was hydrodissected off the vas deferens and vessels and a safe space through which the suture could pass without compromising these structures was created; additionally, high ligation of the hernia sac can be performed at the same time without the need for re-exhalation.

In January 2019, we modified the surgical technique and initiated a novel single-port laparoscopic herniorrhaphy technique for treating paediatric giant inguinal hernia by using double-modified hernia needles and water injection; in the last two years, we have successfully applied this novel operation to more than 106 children with isolated giant inguinal hernias whose inner ring orifice sizes were estimated by the length of the inclined surface of the hernia needle, and the hernia recurrence rate is low. In addition, there was no statistically significant difference in the incidence of complications other than surgical scars between the two groups. Compared with those in the TLPEC group, children in the SLPEC group had shorter operation times, smaller incisions, fewer scars, less pain, and earlier postoperative recovery. Therefore, single-port laparoscopic herniorrhaphy using double-modified hernia needles and water injection to treat paediatric giant inguinal hernia is a safe, effective and minimally invasive surgery.

Our study also has a few limitations. First, this was a single-centre study, and additional research from multiple centres is needed to assess the effectiveness and complications of this technique. Second, this was a retrospective review, and the sample size was limited. Third. only 1 patient experienced relapse in the study, and the difference between the groups was not statistically significant. This situation may be related to the short follow-up time, which is also a minor shortcoming of this study, and a longer follow-up time may be required in the later stage. We will collect follow-up data in a later study to further analyse the possible causes of recurrence. In addition, the follow-up time was too short to compare the effects of the two surgical methods on the children’s reproductive behaviour in adulthood.

## Conclusion

Our study showed that compared with the TLPEC group, the SLPEC group had the advantages of a concealed incision, light scarring, minimal invasiveness, a reduced operation time, minimal bleeding, mild pain and rapid recovery. In conclusion, compared with conventional surgery, single-port laparoscopic herniorrhaphy for treating paediatric giant inguinal hernia through the use of double-modified hernia needles and water injection is a safe, effective and minimally invasive procedure. The cosmetic results are impressive, and the follow-up results are promising.

## Methods

### Study population

The study was reviewed and approved by the institutional ethics board of Fujian Children's Hospital and followed the ethical principles of the 1964 Declaration of Helsinki. All patients’ guardians signed an informed consent form before the operation. We identified 249 patients with isolated giant inguinal hernias who underwent TLPEC or transumbilical SLPEC using double-modified hernia needles with hydrodissection and high ligation of the hernia sac between January 2019 and December 2021. Ultimately, we retrospectively analysed the clinical data of 219 patients with paediatric giant inguinal hernias in our hospital from January 2019 to December 2021, including preoperative, intraoperative, postoperative and follow-up data.

Only patients who presented with giant inguinal hernia and had a history of intermittent incarceration (self-reduction or manual reduction) were included. The inclusion criterion was a large hernia with a diameter of the inner ring opening ≥ 1.5 cm under laparoscopy (using the comparison method between the length of the blunt inclined surface of the hernia needle and the diameter of the inner ring opening during surgery).

Patients were excluded from this study if ① they had other congenital deformities, such as cryptorchidism or hydroceles; ② they had recurrent inguinal hernias; ③ they had sliding hernias; ④ they had serious cardiopulmonary disease or coagulopathy; or ⑤their guardians refused to sign the consent form for surgery or refused to comply with the follow-up schedule.

### Surgical methods

#### Single-port laparoscopic percutaneous extraperitoneal closure using double-modified hernia needles and water injection

**Figure 2:**
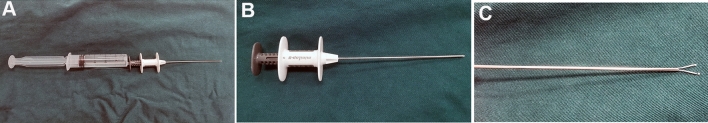
The double-modified hernia needle and water injection (**A**) The double-modified hernia needle and water injection apparatus; (**B**) The double-modified hernia needle ; (**C**) The magnifed image of the two-hooks in the core distal end.

**Figure 3:**
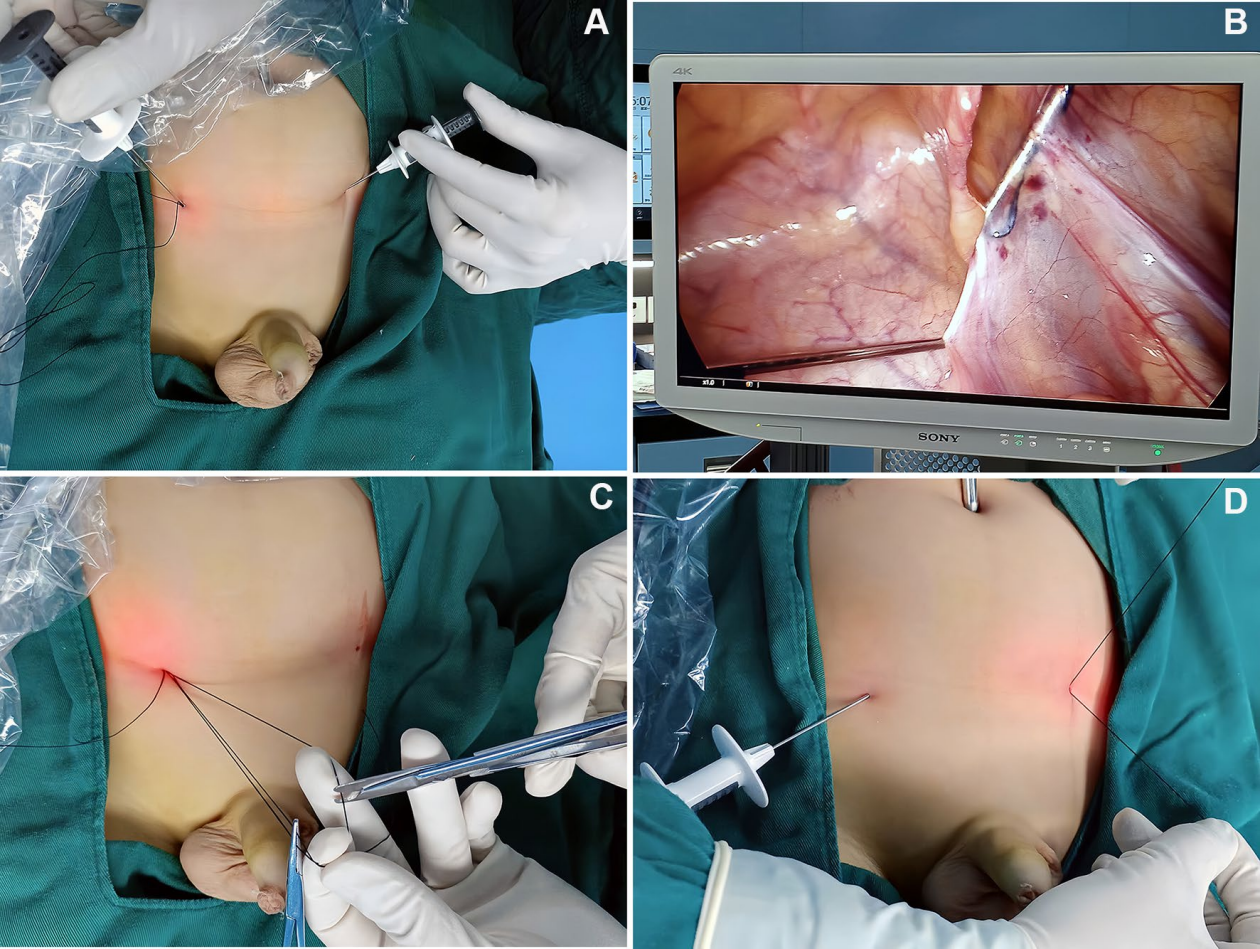
Images of single-port laparoscopic percutaneous extraperitoneal closure with double-modified hernia needles and water injection apparatus. (**A**) The double two-hooked hernia needles and trocha position diagram (**B**) one needle enters the abdominal cavity around the lateral side of the hernia ring and passes between the peritoneum and the vas deferens,the other hernia needle was used to clamp the lateral side of the spermatic cord and acted as an auxiliary clamp to stretch it flat. (**C**) The hernia needle hooks the preset silk thread and takes it out of the abdominal cavity. (**D**) The thread is tightened and tied under the skin to close hernia ring.

In the SLPEC group, laparoscopic percutaneous extraperitoneal closure was performed with double-modified hernia needles (Fig. 2), which were produced by Xiamen Surgaid Medical Equipment Co., Ltd., of China (Patent No. ZL 2013 20013865.2), and water injection. All patients were given general anaesthesia. The patients were asked to urinate and defecate before the operation, and they were placed in a supine position after anaesthesia. A 5-mm diameter trocar was placed on the umbilicus, and a 30° laparoscopic lens was placed. The peritoneum was punctured, and the abdominal pressure was maintained at 8–10 mmHg.After entering the abdominal cavity, the organs were routinely explored, the bilateral internal rings were closed, and the intraoperative inguinal hernia was identified. Under close monitoring under laparoscopy, the skin was punctured with a knife tip or syringe needle at the lower abdominal transverse line corresponding to the inner ring of both sides, and a 2–0 silk thread hook was hung in the shallow groove in front of the two-hooked hernia needle on the affected side. One hernia needle was inserted into the inner ring in the abdominal cavity from the anterior abdominal wall. When the hernia needle reached the vas deferens and spermatic vessels, if the abdominal cavity was very deep or there were many peritoneal folds at the inner orifice and it was difficult to puncture over the deferent duct, the other hernia needle was used to clamp the lateral side of the spermatic cord and acted as an auxiliary clamp to stretch it flat.The surgeon used a syringe connected to the tail to inject normal saline into the extraperitoneal space to separate the extraperitoneal space and reduce damage to the vas deferens and spermatic vessels. With the help of the laparoscopic lens, the silk thread was retained in the abdominal cavity. Then, the hernia needle was slowly retracted into the extraperitoneal space via the original route; however, it could not retreat to the muscular layer and could not be removed from the puncture point. Next, the needle was inserted under the peritoneum on the outside of the inner ring, and the abdominal cavity was entered again through the puncture hole in the peritoneum. The head end of the reserved silk thread in the abdominal cavity was located and removed from the abdominal cavity. Finally, the silk thread was ligated to the hernia sac and tied in vitro with the knot located under the skin (Fig. 3).

#### Two-port laparoscopic percutaneous extraperitoneal closure

In the TLPEC group, the patients were asked to urinate and defecate before the operation, and they were placed in the supine position after anaesthesia. All patients were given general anaesthesia. A 5-mm diameter trocar was placed on the umbilicus, and a 30° laparoscopic lens was placed. The peritoneum was punctured (with the abdominal pressure maintained at 8–10 mmHg). A 3-mm incision was made next to the umbilicus to place grasping forceps for assistance. One hernia needle was inserted into the inner ring in the abdominal cavity from the anterior abdominal wall on the affected side. With the help of the laparoscopic lens and nondamaging pliers, the silk thread was retained in the abdominal cavity. Then, the hernia needle was slowly retracted into the extraperitoneal space via the original route, but it could not retreat to the muscular layer and could not be removed from the puncture point. Next, the needle was inserted under the peritoneum on the outside of the inner ring, and the needle entered the abdominal cavity again through the puncture gap of the peritoneum. The head end of the silk thread reserved in the abdominal cavity was located and removed from the abdominal cavity. Finally, the silk thread was ligated to the hernia sac and tied in vitro, with the knot located under the skin^35^.

#### Protocol for patients who presented with incarceration

If the duration of incarceration was less than 12 h and the child was in good condition, manual reduction was attempted in the nonanaesthetic state. If reduction was not possible and there was no obvious necrosis of the bowel, manual reduction was performed again under direct view via laparoscopy under general anaesthesia. If the patient’s hernia could still not be reduced or the bowel had been obviously damaged, laparotomy was performed, and the patient was not included for evaluation in this study.

### Follow-up schedule

Preoperative and intraoperative clinical data were collected from operative reports and medical records. Postoperative clinical data, which included data on ipsilateral recurrent hernia, pain visual analogue scale (VAS) score, surgical site infection and other surgical complications, were collected. Pain VAS scores were obtained through regular outpatient or inpatient follow-up by telephone^36^. The VAS was used to score postoperative pain at 4 h using a 10-point scale, and the higher the score was, the more pain the patient. All patients were followed up for more than 12 months.

### Statistical analysis

Statistical analysis was conducted using SPSS 19.0 (SPSS Inc., Chicago, IL, USA). The chi-square test was used to compare qualitative variables; other comparisons were made using appropriate statistical tests. Student’s t test was performed to compare continuous variables. Variance analysis was used to compare the mean values between groups. P < 0.05 was considered to indicate statistical significance.

## Data Availability

The data that support the findings of this study are available from the corresponding author upon reasonable request.
